# Preparation and Characterization of Magnetic Polypyrrole Composite Microspheres Decorated with Copper (II) As A Sensing Platform for Electrochemical Detection of Carbamazepine

**DOI:** 10.22037/ijpr.2020.13922.11993

**Published:** 2020

**Authors:** Azadeh Fatahi, Reihaneh Malakooti, Mohsen Shahlaei

**Affiliations:** a *Nanochemistry Research Laboratory, Department of Chemistry, University of Birjand, Birjand, Iran. *; b *Pharmaceutical Sciences Research Center, Department of Medicinal Chemistry, Faculty of Pharmacy, Kermanshah University of Medical Sciences, Kermanshah, Iran.*

**Keywords:** Differential pulse voltammetry (DPV), Carbamazepine, Fe3O4@PPy–CuII composite Microspheres, Electrochemistry

## Abstract

With a facile solvothermal technique, synthesis and application of Fe_3_O_4_@PPy–Cu^II^composite microspheres in the carbon ionic liquid matrix have been reported as highly sensitive sensors for voltammetric determination of Carbamazepine (CBZ). The morphology, crystal phase, and structure of synthesized nanocomposite were confirmed by routine methods, such as transmission electron microscopy (TEM), scanning electron microscopy (SEM), X-ray photoelectron spectroscopy (XPS), X-ray diffraction (XRD), Fourier translation infrared spectroscopy (FT-IR), thermal gravimetric analysis (TGA), and inductively coupled plasma atomic emission spectroscopy (ICP-AES). Under the optimized conditions, differential pulse voltammetric (DPV) peak current was proportional to the CBZ concentration in the range of 0.05 to 25 μM with the detection limit (S/N = 3) of 32 nM. The storage stability of the modified electrode was also investigated which shows that the current responses remain about 95.2% of their initial values, indicating the appreciable storage stability of this sensor.The proposed electrode displayed excellent repeatability and it was satisfactorily used for determination of CBZ in real samples (urine, and serum samples) with high recovery.

## Introduction

Carbamazepine (CBZ) is one of the most popular pharmaceuticals used for the treatment of epilepsy and bipolar disorder (1). Carbamazepine is one of the most extremely prescribed drugs and due to its high rate of consumption in today’s society, it is essential to develop and establish new, quick, and accurate analytical techniques for the determination of this drug (2). Until now, several analytical methods have been reported in literature for determination of CBZ such as high performance liquid chromatography (3, 4), gas chromatography (5), capillary chromatography (6), mass spectrometry (7), chemiluminescence (8) and spectrophotometry (9). Although these analytical techniques are associated with the relative advantages of sensitivity and accuracy, they require fastidious sample preparation and need relatively expensive equipment, which consequently increase the time and cost of analysis. In this regard, the determination of electroactive species using electroanalytical techniques with respect to their advantages such as easy operation and fast response, less consumption of reagents, low cost, more sensitivity and high selectivity, have received considerable attention in recent years (10-13). 

For the electrochemical determination of CBZ using the modified electrode, it must be noted that the modifier is one of the most important and influencing factors that can heavily affect the determination sensitivity and selectivity. 

In recent years, a karge number of attempts have been made to synthesize the nano-sized materials in different fields of science and technology. Among different nanomaterials, metallic nanoparticles with their high surface reaction activity, high catalytic efficiency, strong adsorption ability and unique ability to promote fast electron transfer between electrode and target were expected to be a promising candidate for the design of electrochemical sensors (14). 

Among these nanoparticles, Fe_3_O_4_ nanoparticles have been widely used in electrochemical sensing due to their unique properties such as low-cost, large surface-to-volume ratio, low toxicity and easy-preparation (15). The embedment of metallic magnetic NPs cores inside of conducting polymers, such as polypyrrole (PPy) can improve the physical and chemical properties of polymeres because of the strong electronic interaction between the NPs and the polymer matrices (16-18). Conducting polymer has been extensively studied for utilizing as the main part of chemical sensors and biosensors (19-24). Among them polypyrrole (PPy) is a kind of high electrical conductive, good biocompatible, and ease synthesis. Simultaneously, PPy can support in obtaining a good dispersion of metal nanoparticles due to the intrinsic existence of functional groups within long carbon chains (25, 26). On the other hand, metal doped metal oxide is receiving considerable interest because of their excellent physical and catalytic properties of these materials (27). Copper (Cu) based materials, such as copper bulk metal, copper nanoparticles (CuNPs), and copper complexes have attracted considerable interest as sensors and biosensors due to their distinct advantages of environmental benignity, low cost and high electrical conductivity and the possibility of promoting electron transfer reactions at a lower over potential (28). Furthermore, Cu loaded onto Fe_3_O_4_ improve the catalytic activity due to the synergetic effect (29).

Carbon ionic liquid electrode (CILE), as a relatively new branch in sensors, would construct by incorporating ionic liquids in the traditional CPE. The electrochemical performance of CILE can be greatly enhanced by adding ionic liquids into the CPE, which was owing to the excellent electrochemical properties of ionic liquids, such as wide electrochemical windows, negligible vapor pressure, excellent *thermal* and electrochemicall stability, high intrinsic conductivity,* etc. *(30). 

In this research, the synthesis of Fe_3_O_4_@PPy–Cu^II^composite microspheres is reported with well-defined morphology. Then, a novel electrochemical sensor was fabricated based on a CILE for the electrochemical detection of CBZ. The results show that combination of unique properties of ionic liquids and electrocatalytic activity of Fe_3_O_4_@PPy–Cu^II^composite microspheres, lead to new sensing surface for voltammetric determination of CBZ with good sensitivity, acceptable selectivity, and low detection limit.

## Experimental

All electrochemical experiments were performed using a potentiostat-galvanostat Autolab equipped with a three-electrode configuration containing a saturated calomel electrodeas a reference electrode and the auxiliary electrode wasa platinum electrode. CILE modified with Fe_3_O_4_@PPy–Cu^II ^composite microspheres was applied as working electrode. The system was run on a PC by NOVA and FRA 1.11 software. The synthesized Fe_3_O_4_@PPy–Cu^II ^composite microspheres, characterized by Power X-ray diffraction (XRD) patterns were obtained with a Bruker D8-advance X-ray diffractometer which the wavelength of x-ray was 0.154 nm (Cu K-alpha) and Thermo gravimetric analysis (TGA) were carried out with a Shimadzu thermo gravimetric analyzer (TG-50). This system was equipped with a concentric hemispherical (CHA) electron energy analyzer (Specs model EA10 plus) suitable for X-ray photoelectron spectroscopy (XPS). ICP analysis was performed using OPTIMA 7300 DV ICP analyzer. Transmission electron microscopy (TEM) analysis was performed using a (Philips CM30). Fourier transform infrared (FTIR) spectra of KBr powder pressed pellets were recorded on a Shimadzu FTIR*-*8300*.* The morphology of the products was studied by using Hitachi Japan, model s4160 Scanning Electron Microscopy (SEM). All chemicals used in this work were analytical grade, Merckand Fluka Chemical Company. PH studies were conducted in Britton–Robinson (B–R) buffer solutions, consisting of a mixture of acetic acid, boric acid and phosphoric acid solutions. CBZ powder (pure) was purchased from Aldrich chemicals (Milwaukee, USA). All the reagents used were of analytical grade and double distilled water was used throughout the experiments.

*Synthesis of Fe*_3_ *O*_4_ *microspheres*

Magnetite particles were prepared by using a solvothermal method (31). The details were as follows: anhydrous ferric chloride hexahydrate (FeCl_3_·6H_2_O) (1.4 g, 5.2 mmol) and Na_3_Cit (0.29 g, 0.96 mmol) were dissolved in EG/ethanol (36 mL/4 mL) solution with stirring to form a clear solution; then, NaAc (1.9 g, 23 mmol) was added under vigorous stirring for 5 min. The obtained yellow solution was then transferred to a Teflon-lined stainless steel autoclave (with a capacity of 50 mL) for heating at 200 ◦C for 10 h. Then, the autoclave was carefully taken out and allowed to cool down to room temperature. The as-made black products were thoroughly washed with ethanol for three times, and they were then vacuum-dried.

*Synthesis of Fe*_3_ *O*_4_ *@PPy composite microspheres*

Fe_3_O_4_ microspheres (0.3 g) were dispersed in H_2_O (70 mL) with sonication. Subsequently, pyrrole (3 mL) in methanol (15 mL) and HCl solution (15 mL, 6 M) were added into the above solution, and sonicated for 1.5 h to produce the PPy coating on the Fe_3_O_4_ microspheres. Finally, product of the obtained particles was washed with methanol and deionized water to remove the residual pyrrole monomers and HCl acid, and dried in a vacuum at 60 *°*C for 24 h *(*32).

*Loading of Cu on Fe*_3_ *O*_4_ *@PPy* *(Fe*_3_*O*_4_*@PPy–Cu*^II^*)*

The as-synthesisedFe_3_O_4_@PPy microspheres (100 mg) were first dispersed in ethanol solution (50 mL) during 0.5 h under) under ultrasonication. The formed black suspension mixed with 30 mL, 0.1 m of CuCl_2_ under ultrasonic for 1 h. Finally, the microspheres were separated and collected with a magnetand washed with deionized water for several times and dried under vacuum.

*Preparation of modified electrode*

Several electrodes with different percent of the Fe_3_O_4_@PPy–Cu^II^composite microspheres, graphite powder, paraffin and solid 1-Ethyl-3-methylimidazolium hexafluorophosphate (EMIMPF_6_) were prepared and examined for simultaneous determination of CBZ under identical conditions. The maximum sensitivity was obtained when the amounts of the graphite powder, Fe_3_O_4_@PPy–Cu^II^, paraffin oil, and ion liquid EMIMPF_6_ in the paste were 60:15:10:15% (w/w). Then, the mixture was mixed well in an agate mortar and ground into a homogeneous paste. The paste was packed into pipette tube and a copper wire was utilized for electrical connection. The bare carbon paste electrode CPE was fabricated by mixing 30.0 w/w% of paraffin oil and 70.0 w/w% of graphite powder. The CILE was fabricated by mixing 20.0 w/w% of paraffin oil, 15.0 w/w% of solid (EMIMPF_6_), and 65.0 w/w% of graphite powder.

*Preparation of real samples*

In order to demonstrate the application of the developed electrochemical sensor for determination of CBZ, human serum and urine samples were used. Human urine and serum samples were taken from healthy donors and stored in a refrigerator at 4 ℃ immediately after collection. The Blood serum sample was deproteinized by adding 2 mL saturated ammonium sulfate solution to 10 mL sample and the solution was centrifuged then the sample was diluted 10 times with PBS buffer (pH 7.0) then appropriate amounts of this diluted sample was transferred to the electrochemical cell. The Urine sample was centrifuged and diluted 10 times without any further pretreatment. Since the matrix of samples is also so complicated, the CBZ determination was not possible in this study and therefore, CBZ was spiked to the blood and urine samples at three concentrations of 3, 5, and 10 μM. The total CBZ content of real samples was then determined using standard addition method.

## Results and Discussion

*The preparation of of Fe*_3_ *O*_4_ *@PPy–Cu* ^II^ *composite microspheres*

**Scheme 1:**
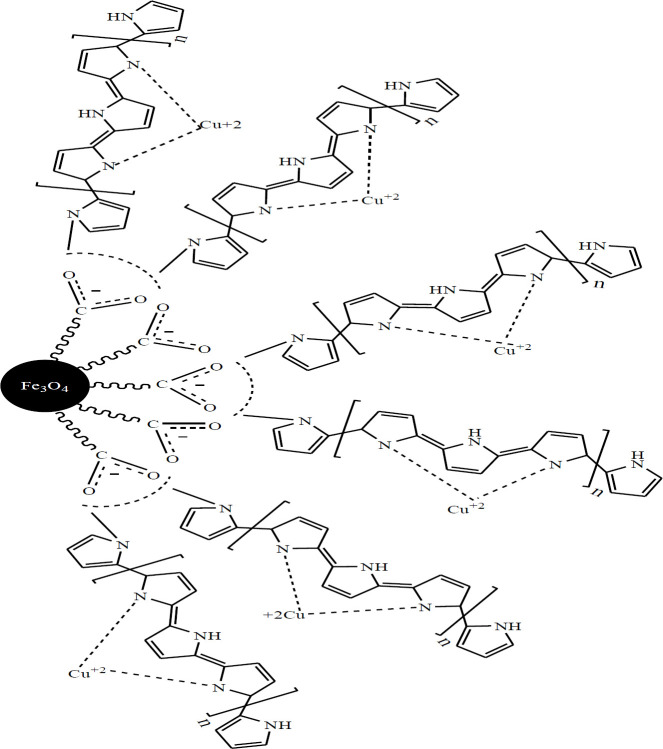
The preparation process of Fe_3_O_4_@PPy–Cu^II^

**Figure 1:**
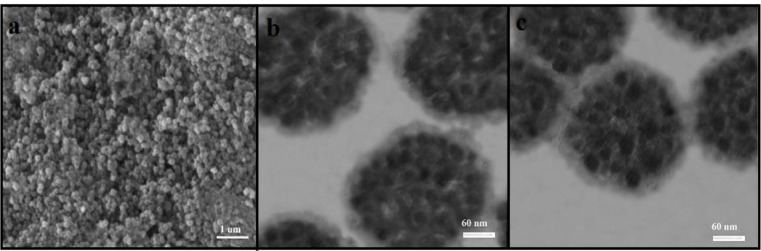
(a) SEM images of Fe_3_O_4_; (b) TEM images of Fe_3_O_4_@PPy; (c) TEM images of Fe_3_O_4_@PPy–Cu^II^

The preparation plan of Fe_3_O_4_@PPy–Cu^II^composite microspheres was shown in Scheme 1. .Figure 1a is the scanning electron microscope (SEM) image of the Fe_3_O_4_ microspheres. As it can be seen that the as-prepared Fe_3_O_4 _microspheres have a spherical shape with an average diameter of 140-145 nm. The Fe_3_O_4_@PPy is composed of microspheres with a mean diameter of 300 nm, and a continuous layer of PPy can be observed on the outer shell of the Fe_3_O_4_ microsphere and the thickness of these shells are about 20 nm (Figure 1b). In Figure 1c, it could be seen that the morphology of Fe_3_O_4_@PPy–Cu^II ^almost remained the same after addition of CuCl_2_ on Fe_3_O_4_@PPy composite microspheres*.*

**Figure 2:**
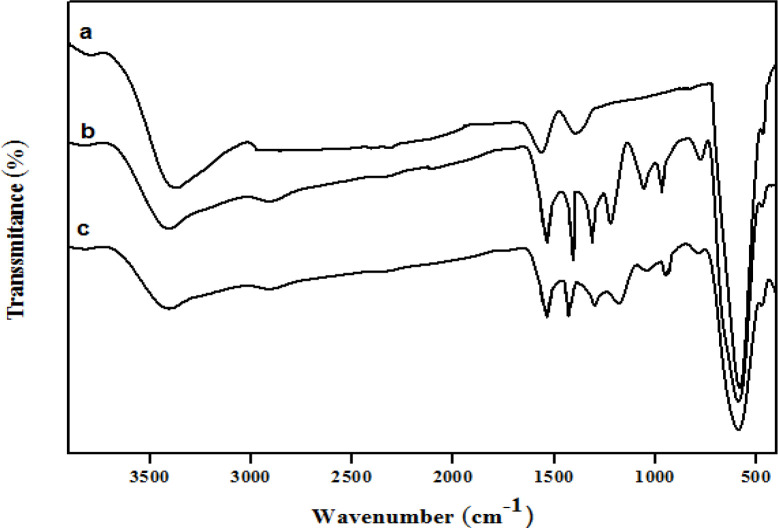
FT-IR spectra of (a) Fe_3_O_4_ (b) Fe_3_O_4_@PPy and, (c) Fe_3_O_4_@PPy–Cu^II^.

The structures of the Fe_3_O_4_ microspheres, Fe_3_O_4_@PPy, and Fe_3_O_4_@PPy–Cu^II^ were analyzed using FT-IR spectroscopy, as shown in Figure 2, respectively. In curve (a), the strong absorption peak at 576 cm^-1^ corresponds to the Fe–O vibrations, the adsorption peaks were located at 3384, 1622, and 1406 cm^-1^ and can be attributed to the stretching vibration of -OH, C**=**O, and C-O of carboxyl groups, respectively. In the Fe_3_O_4_@PPy spectrum (Figure 2b), peaks at 1559 cm^-1 ^and 1453 cm^-1^were assigned to the characteristic absorption peaks of pyrrole rings (33). Furthermore, the peaks at 1332 cm^-1^, 1049 cm^-1^, and 934 cm^-1 ^could be attributed to C–N in-plane (34), N–H in-plane (35) and C=C out-of-plane deformation vibrations (33) in the pyrrole ring, respectively. The bands at 1162 and 775 cm^−1^ corresponded to C–H in-plane and out-plane vibration of pyrrole (36). The FT-IR spectrum of Fe_3_O_4_@PPy–Cu^II^ (Figure 2b) was similar to that of Fe_3_O_4_@PPy, but the C–N stretching frequency shifted to a lower wavenumber, which was probably caused by N bonded to electron-deficient Cu to form the Cu complex (37).

**Figure 3:**
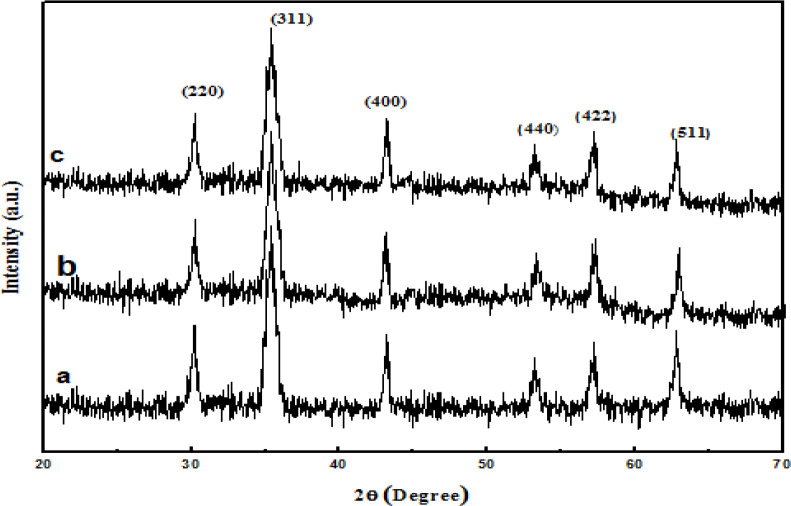
XRD patterns of (a) Fe_3_O_4_; (b) Fe_3_O_4_@PPy and (c) Fe_3_O_4_@PPy–Cu^II^.

XRD analysis of the samples was performed Figure 3 all detected diffraction peaks marked in Figure 3a can be indexed to (111), (220), (311), (400), (422), (511), (440), and (533) planes of face-centered cubic Fe_3_O_4_ phase. In Figures 3b and3c, the main peaks of Fe_3_O_4_@PPy, Fe_3_O_4_@PPy–Cu^II are similar to the Fe^_3_O_4_. Thus, either the coating of PPy shell or the immobilizing Cu (II) does not affect the crystal structure of the Fe_3_O_4_ particles. 

**Figure 4:**
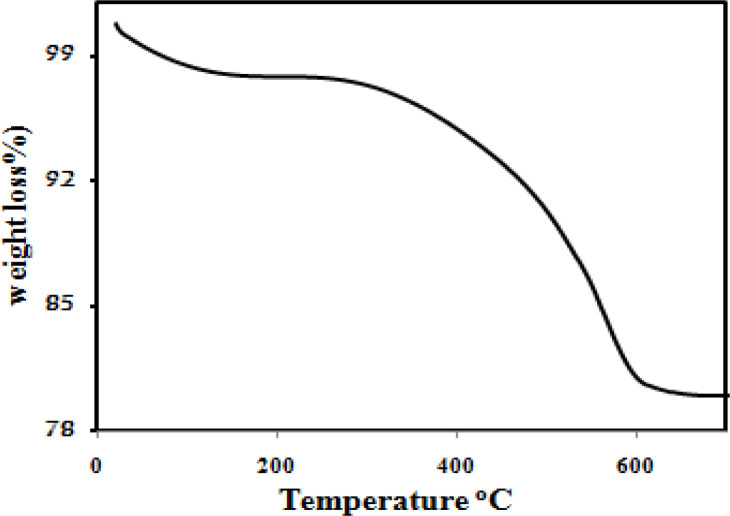
TGA curve of the as-prepared Fe_3_O_4_@PPy–Cu^II^ microspheres

Figure 4 illustrates the results of the thermogravimetric analysis of the Fe_3_O_4_@PPy–Cu^II^. The initial mass loss at lower temperatures was assigned to the release of water and solvent molecules in the polymer matrix. A sharp decrease in mass was observed at 300 °C due to thermal degradation of the PPy chains. From the TG analysis, the mass percentages of the PPy in the magnetic core/shell composite is about 18%.

**Figure 5:**
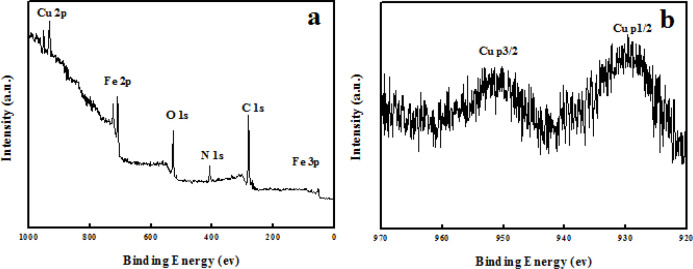
XPS spectra of (a) Fe_3_O_4_@PPy–Cu^II^; (b) Cu 2p of Fe_3_O_4_@PPy–Cu^II^

The copper content in Fe_3_O_4_@PPy–Cu^II ^composite microspheres was determined by means of ICP-AES and amounted to 4 wt%. The X-ray photoelectron spectroscopy (XPS) elemental survey scans of the surface of Fe_3_O_4_@PPy–Cu^II^ show clear peaks corresponding to oxygen, nitrogen, carbon, copper, and iron which confirm the successful formation of Fe_3_O_4_@PPy–Cu^II ^composite microspheres (Figure 5a). As it can be observed, in Figure 5b the peaks located at 933 and 952.8 eV are attributed to the core level Cu 2p_3/2 _and Cu 2p_1/2_, respectively, which confirms that the oxidation state of copper in the Fe_3_O_4_@PPy–CuIImicrospheres is (+ II) (38).

**Figure 6:**
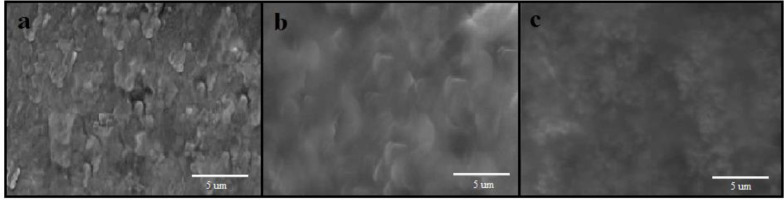
SEM images of: (a) CPE, (b), CILE and (c) Fe_3_O_4_@PPy–Cu^II^/CILE

The typical SEM images of different electrodes were shown in Figure 6 As can be seen at the surface of a bare CPE (Figure 6a), irregularly shaped graphite flakes and separated layers were isolated from each other. As seen in Figure 6b, the SEM image shows CPE sheets in presence of ionic liquid more uniform and smooth without separated carbon layer, which was due to binding and blanketing role of ionic liquid. After Fe_3_O_4_@PPy–Cu^II^ were added to carbon ionic liquid (Figure 6c), it can be seen that they were distributed on the surface of electrode with spherical structure.

**Figure 7:**
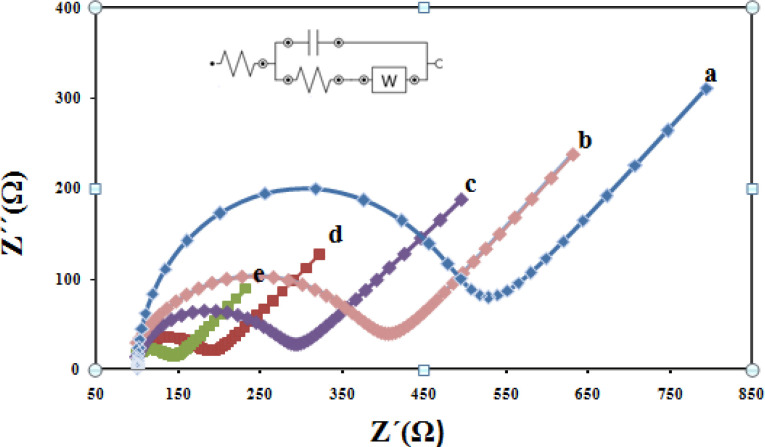
EIS for (a) CPE, (b) CILE (c) Fe_3_O_4_/CILE (d) Fe_3_O_4_@PPy /CILE/ and Fe_3_O_4_@PPy–Cu^II^/CILE (e) in 1 mM (Fe(CN)_6_)^3-/4- ^with 0.1 M KCl

For further electrochemical characterization, Nyquist plots of CPE, CILE, Fe_3_O_4_/CILE, Fe_3_O_4_@PPy /CILE, and Fe_3_O_4_@PPy–Cu^II^/CILE were recorded in the presence of 1.0 mM(Fe(CN)_6_) ^3-/4-^ with 0.1 M KCl as the supporting electrolyte are given in Figure 7 As it can be seen, a well-shaped semi-circle was observed at higher frequencies at the CPE, due to the charge transfer process in the electrode–electrolyte interface (curve a). The small charge transfer resistance of CILE in comparison with the bare CPE indicating the presence of high ionic conductive ionic liquids in the carbon paste could greatly enhance the conductivity of the electrode (curve b). From Nyquist plots, the electron transfers resistance value relating to Fe_3_O_4_/CILE is smaller compared with the CILE, because the electrode modified with Fe_3_O_4_ possesses the least electroactive surface area between the above electrodes which is due to the repulsion between *(*Fe (CN)_6_) ^3-/4^^- ^redox probe and negative surface charges of nanoparticles as well as relatively agglomerated nature of nanoparticles themselves (curve c). When Fe_3_O_4_@PPy was incorporated in the electrode, the charge transfer resistance increases dramatically (curve d) due to high conductivity of the PPy polymer. Finally, From Nyquist plots, Fe_3_O_4_@PPy–Cu^II^/CILE show the lowest charge-transfer resistance among the studied five electrodes. This behavior can be attributed to the fact that Cu^2+^^ions^ can facilitate the electron transfer between the electrochemical probe (Fe (CN)_6_) ^3-/4- ^and the electrode surface (curve e).

*Determination of surface area*

**Figure 8:**
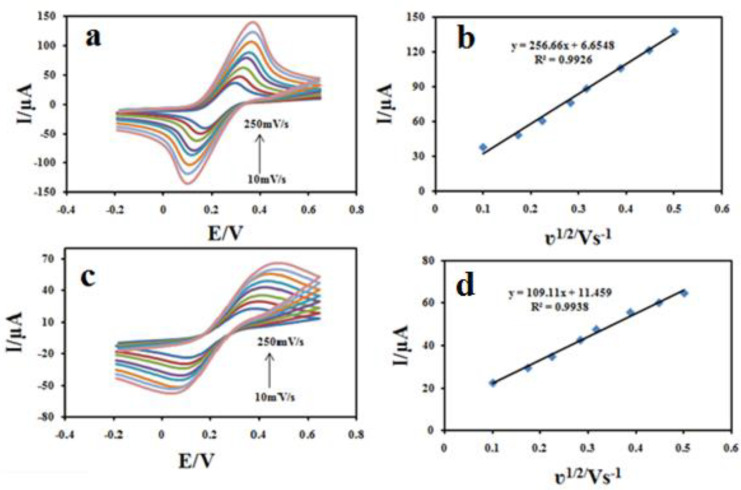
Cyclic voltammograms s of 1 mM (Fe(CN)_6 _)^3-/4- ^in 0.1 M KCl at various scan rates (a-i) (10-250 mV s^-1^) on Fe_3_O_4_@PPy–Cu^II^/CILE (a) and CILE (c); The slope of Ipa *vs. *v^1/2^ for 1 mM (Fe(CN)_6 _)^3-/4-^ on Fe_3_O_4_@PPy–Cu^II^ /CILE (b) and CILE (d).

To illustrate that the Fe_3_O_4_@PPy–Cu^II ^could improve the surface area of the CILE, the electroactive surface areas (A) of ordinary CILE and Fe_3_O_4_@PPy–Cu^II ^modified electrodes were determined using CV in a 1.0 mM (Fe(CN)_6_) ^3-/4-^solution containing 0.1 M KCl at different scan rates (v) according to the Randles-Sevcik Equation (39), as follows: ip = 2.69 × 10^5 ^A D^1/2 ^n^3/2 ^v^1/2^ C, where Ip is the peak current, D is diffusion coefficient (7.6 × 10^–6^ cm^2^s^–1^),v is scan rate (Vs^–1^) , C_0_ is the concentration of K_4_(Fe(CN)_6_) in mol L^–1^, υ is the scan rate *n* is the number of electron transferred, and A is the effective surface area. As shown in Figure 8, both the peak currents (ip) of Fe_3_O_4_@PPy–Cu^II^modified electrode (Figure 8a) and unmodified electrodes (Figure 8b) were proportional to the square root of the scan rate. The surface area could be calculated from the slope of ip *vs* v^1/2^ plot, which were found as 0.147 cm^2^, and 0.376 cm^2^ for bare CILE and Fe_3_O_4_@PPy–Cu^II^/CILE, respectively, where the electroactive area of the electrode increased 2.49 fold. The results show that the presence of Fe3O4@PPy–Cu^II^ makes the active surface of the electrode larger.

*Electrochemical behavior of CBZ at Fe*_3_ *O*_4_ *@PPy–Cu* ^II^ */CILE*

**Figure 9:**
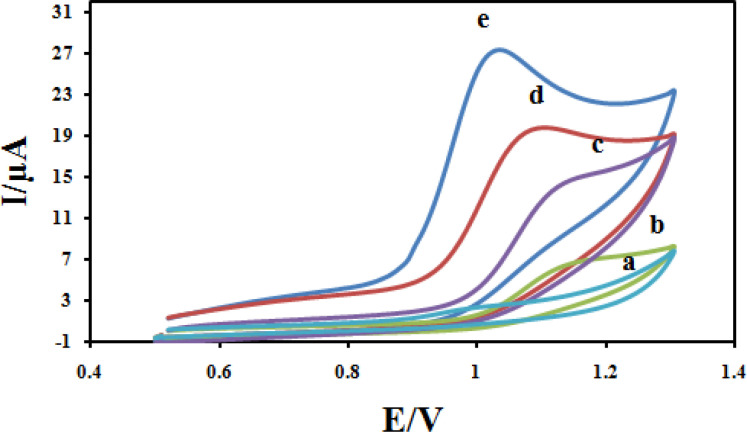
Cyclic voltammograms for 100 μM CBZ at scan rate 100 mV s^-1^ at CPE (a); CILE (b); Fe_3_O_4_/CILE (c); Fe_3_O_4_@PPy/CILE (d) and Fe_3_O_4_@PPy–Cu^II^/CILE (e) in B-R buffer

The electrochemical behavior of CBZ at different modified electrodes was studied in 0.04M solution of Britton–Robinson buffer (BR) and a 100 μM CBZ solution with a cyclic voltammetry technique, and the results are shown in Figure 9 Due to slow electron transfer, CBZ did not show obvious oxidation peak at bare CPE (curve a), while the responses were considerably improved at the CILE (curves b), As can be seen in curves c, the peak current increased due to the presence of Fe_3_O_4 could increase_ active surface area. The significant increase in peak current and shift in peak potential at the surface of Fe_3_O_4_@PPy/CILE in comparison with those obtained CILE/Fe_3_O_4_, CILE and CPE is due to the large surface and high conductivity of Fe_3_O_4_@PPy. It also must be noted that the enhancement in the peak currents and shift in peak potential towards less positive potentials at the surface of Fe_3_O_4_@PPy–Cu^II^/CILE in comparison with Fe_3_O_4_@PPy/CILE, indicates the better catalytic activity of Fe_3_O_4_@PPy–Cu^II^due to synergism effect of copper ions in the composition of the modified electrode.

*Investigation of the scan rate*

**Figure 10:**
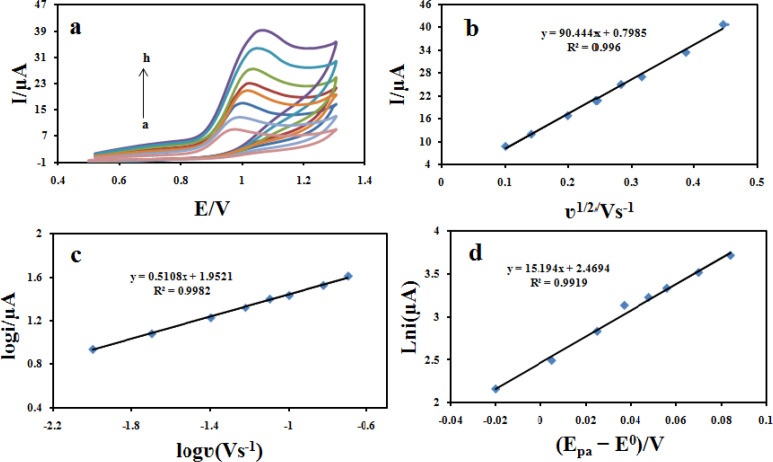
(a) Cyclic voltammograms of 100 μM CBZ at Fe_3_O_4_@PPy–Cu^II^/CILE in B-R buffer at different scan rates. The numbers of 1–8 correspond to 10, 20, 40, 60, 80, 100, 150 and 200 mVs^−1^, respectively. (b) Variation of the peak current with square root of scan rate (υ^ 1/2^); (c) Variation of the the logarithmic peak currents vs. the logarithmic scan rate, and (d) plot of (E_pa_–E^0^) and the logarithmic scan rate

The influence of potential scan rate on the oxidation reaction of 100 μM CBZ at the Fe_3_O_4_@PPy–Cu^II^/CILE was investigated by cyclic voltammetry (Figure 10a). The results showed that the peak currents vary linearly with the square root of the scan rate (υ^1/2^) (Figure 10b), which confirms a diffusion-controlled process for CBZ oxidation on the surface of the modified electrode in the studied range of potential sweep rates, with following Equations: I_pa_ = 90.444 υ^1/2^ +0.7958 (R^2^ = 0.996). A plot of log i_pa_ *vs*. log υ also yields a straight line (Figure 10C) with the linear regression Equation of log i_pa_ (μA) = 0.5108 log υ (V s^-1^) + 1.9521 (R^2 ^= 0.9982). The slope of 0.51 is very close to the theoretical value of 0.5, which further confirms a diffusion-controlled process in this case.

*Charge transfer coefficient*

The transfer coefficient (α), is a quantity which characterizes the effect of electrochemical potential on the activation energy of an electrochemical reaction, can be obtained using Equation 1 for an irreversible diffusion-controlled process. This Equation refers to peak potential and natural logarithm of peak current (ln i_pa_), and can be expressed as follows (40): 

I_pa_ = 0.227 nFAC × k_s _exp (-α F/ RT (E_pa_− E^0^)) 

(Equation 1)

where α is the electron transfer coefficient, n is the number of transferred electrons, k_s_ is the heterogeneous electron transfer rate constant, E^0^ is the formal redox potential and the other symbols have their usual meanings. The value of E_0_ in Equation 1 can be obtained from the intercept of Ep *vs.* curve by extrapolating to the vertical axis at = 0 when ν was approached to zero (41). Moreover, value of α can be calculated from the slope the dependence of ln i_pa_ on (E_pa_-E^0^) (Figure 10d). With the slope of 15.543, the value of α was evaluated to be 0.38 for the Fe_3_O_4_@PPy–Cu^II^/CILE, which is smaller than the reported value of 0.49 at ERGO–SWCNT modified GCEs (42). The lower α value suggests that the oxidation of CBZ proceeds more easily at Fe_3_O_4_@PPy–Cu^II^/CILE, for which a reasonable reason is that the better conductivity and lots of defect sites of Fe_3_O_4_@PPy–Cu^II^ hybrid facilitate the electrons to transfer.

*Diffusion coefficient (D)*

**Figure 11:**
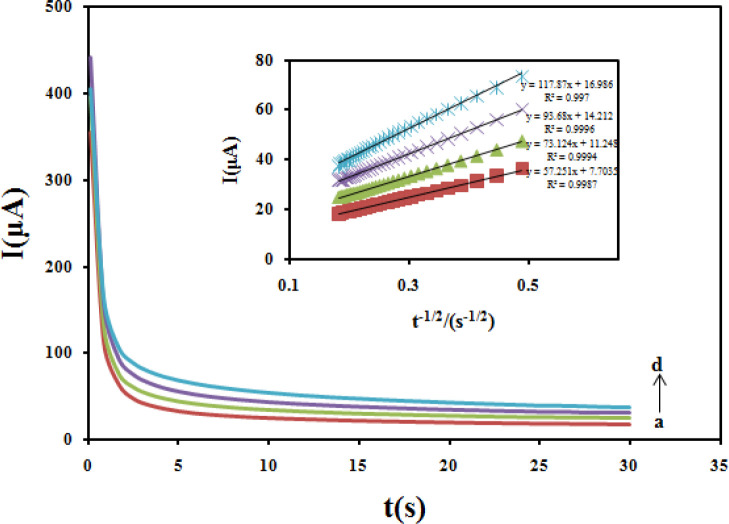
Chronoamperograms obtained at the Fe_3_O_4_@PPy–Cu^II^/CILE in the presence of 300, 400, 500 and 600 μM CBZ in in B-R buffer. Inset) Cottrell's plot for the data from the chronoamperograms

Chronoamperometric method was employed to evaluate the diffusion coefficient (D) of CBZ at the modified electrode (Figure 11). In chronoamperometry studies, the value of D of CBZ was determined in solution by using the Cottrell Equation (43).

I = nFAD^1/2 ^C_0_ π^-1/2^ t^-1/2^

Under diffusion control, a plot of I versus t^-1/2^ will be linear, and from the slope the mean value of D can be determined. The value of the D for CBZ was 2.1 × 10^−5^cm^2^/sec.

*Electron transfer number (n)*

The number of transferred electrons involved in the overall oxidation process (n) and in the rate-determining step (nα) can be calculated from Equation 2 for a totally irreversible diffusion controlled process, ip is defined by the following Equation (44). 

ip = 2.99 × 10^5 ^n ((1-α) nα)^1/2^ v^1/2^ C_0_ A D^1/2^

 (Equation 2)

Where D is the diffusion coefficient (2.1 × 10^−5^cm^2^/s), A is the electroactive area (0.376 cm^2^), and C_0_ (100 μM) is the bulk concentration of CBZ. The linear Equation between i_pa_ and v^1/2^ has been peresented when the scan rate effect was studied. So, the value of n (nα^1/2^) was calculated (2.23) by substituting all the values in Equation 2. Considering the integer value for electron transfer number, the result of n = 2 should be acquired. In addition, the equal numbers of protons and electrons are involved in this electro-oxidation reaction, which are supported by the discussion in the study of effect of pH. Thus, two-proton transferred before or after the rate-determining step.

*Standard heterogeneous rate constant (k*_s_ *)*

The standard heterogeneous rate constant (k_s_) for the totally irreversible oxidation of CBZ can be estimated according to the following Equation provided by Velasco (45):

k_s_ = 2.415 exp (-0.02F/ RT) D^1/2 ^(E_p_–E_P/2_)^-1/2^ υ^1/2^

 (Equation 3)

where E_p_ is the peak potential in mV, and E_P/2_ is the potential where the current equals half of the peak current, also in mV. According to CV curves in Figure 9, E_p_–E_P/2 _is 48 mV. Therefore, the k_s _value of Fe_3_O_4_@PPy–Cu^II^/CILE is 7.33 10^-3^ cm sec^-1^.

*The influence of pH*

**Figure 12:**
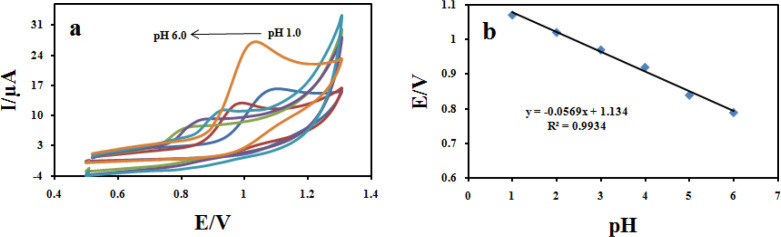
Cyclic voltammograms of 100 μM CBZ at Fe_3_O_4_@PPy–Cu^II^/CILE in B-R buffer recorded (a) from pH 1.0 to 6.0 at a scan rate of 100 mVs^−1^ (b) effect of pH of CBZ solutions on peak potential

The pH of the supporting electrolyte affects the electrochemical behavior of CBZ. To investigate the influence of the pH on the electro-oxidation of CBZ at the Fe_3_O_4_@PPy–Cu^II^/CILE modified electrode, cyclic voltammograms of 100 μM CBZ was recorded at different pH values in the range of 1.0 to 6.0 (Figure 12a). It was found that with the increase in pH of the solution the peak potential shifted negatively suggesting the participation of H^+^ ions in the oxidation reaction. The plot of E_pa_ *vs *pH values show good linear relationships described by the following Equation:

 Epa (V) = −0.056pH + 1.2348 (R^2^ = 0.993) (Figure 12b).

On the other hand, by increasing the pH values, the anodic peak current of CBZ gradually decreased. Hence, Britton–Robinson buffer (BR) solution of pH 2.0 was chosen as the optimum supporting electrolyte and used for all further experiments. The absolute value of the slope 0.05 V pH^−1^ is close to the theoretical value of 0.0586 V pH^−1^ which indicates that the number of protons and transferred electrons involved in the electro-oxidation mechanism is equal (46).

*Analytical characteristics*

The validation tests were carried out for the developed sensor using Fe_3_O_4_@PPy–Cu^II ^composite microspheres. Optimal conditions were used to find out recovery, linearity, correlation coefficient (R^2^), limit of detection (LOD), and intra-day and inter-day precisions of this method.

**Figure 13:**
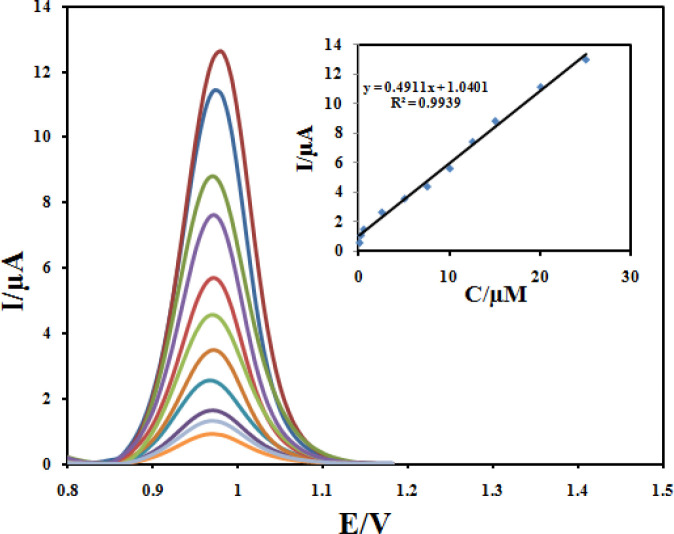
Differential pulse voltammograms of Fe_3_O_4_@PPy–Cu^II^/CILE in B-R buffer containing different concentrations of CBZ in the ranges 0.05–25 μM. Inset: Plot of the peak current against concentration of CBZ

The quantitative determination of CBZ at the Fe_3_O_4_@PPy–Cu^II^/CILE under optimum conditions described above was achieved by DPV. As it can be seen in Figure 13, the oxidation peak current was linearly related to the CBZ concentration in the range of 0.05 to 25 µmol L^-1^ with a calibration Equation of I_p_ = 0.4911C +1.0401 (R^2 ^= 0.9939). The detection limit was estimated to be 0.032 µmol L^-1^.In order to evaluate the analytical performance of the developed sensor, a literature comparison for CBZ determinations using different modified electrodes is shown in Table 1. Clearly, that the Fe_3_O_4_@PPy–Cu^II^/CILE is comparable and even better than those obtained from most of other works with respect to their detection limit and linear dynamic range.

Precision, expressed as relative standard deviation (RSD), was evaluated in terms of repeatability and reproducibility whose value for intra-day RSD% was between 2.3% and 3.9% and for inter-day RSD% was in the range of 4.1–5.1%.

*Real sample and interference study*

The determination of CBZ is clinically significant in therapeutic drug monitoring as it decreases the risk of toxic reactions and increases the possibility of reaching the expected therapeutic result. The practical applicability of the proposed sensor was examined to determine CBZ in human serum and urine samples using standard addition method. The samples were prepared as described in the experimental section. According to the results in Table 2, the good accuracy and precision obtained demonstrate the reliability of the designed sensor for the determination of CBZ in biological fluids. In order to investigate the selectivity of the designed sensor toward CBZ detection, some ordinary compounds in biological media and drugs were selected. Under the optimum conditions, no interference effect was found for the detection of 50 µM CBZ from the following compounds: NaCl, KNO_3_, Tryptophan, Cysteine, Uric acid, and Ascorbic acid.

*Investigation of stability and reproducibility*

The storage stability of the modified electrode was also investigated by examining its response current after storage period of 30 days. It is found that the current responses remain about 95.2% of their initial values, indicating the appreciable storage stability of this sensor. In continuous, the reproducibility of the modified electrode was investigated using five different electrodes. The relative standard deviations (RSD) for peak currents were less than 5.4%, which indicates that reproducibility of Fe_3_O_4_@PPy–Cu^II^/CILE sensor is suitable.

**Table 1:** A comparison of analysis parameters of CBZ with recently reported reference

**Reference**	**LOD** **(μM)**	**Linear** **range (μM)**	**Electrode**
	3.03	5–100	Au/graphene–AuNPs/GCE
	0.029	0.05–3	ERGO–SWCNT/GCE
	0.092	0.5–100	Fe–SnO2/SPCE
	0.04	0.05–100	MWCNT/GCE
	0.016	0.15-100	Fullerene-C60/GCE
	3.89	84.6–846	Graphite/GCE
This work	0.032	0.05 – 30	Fe_3_O_4_/PANI–Cu^II^/CILE

**Table 2:** Determination of CBZ in body fluids using the proposed method

**RSD (%)**	**Recovery (%)**	**Found (μM**)	**Added (μM)**	
-	-	Not detected	0	**Blood serum** **Samples**
1.6	101	3.05 ± 0.05	3	
2.7	102	5.1 ± 0.14	5	
3.2	97.5	9.75 ± 0.32	10	
	-	Not detected	0	**Urine**
2.8	95	2.85 ± 0.07	3	
2.2	101	4.9 ± 0.11	5	
2.3	104	10.2 ± 0.24	10	

## Conclusion

In this study, a synthesis of Fe_3_O_4_@PPy–Cu^II^/CILE composite microspheres were reported and then characterized by TEM, XRD, ICP, TGA, FTIR and XPS techniques. The results showed good stability as well as excellent electro catalytic activity effect for the oxidation of CBZ. The modified electrode exhibited excellent analytical performances such as wide linear range, low detection limit and good selectivity for determination of CBZ. Finally, the novel sensor was applied to the determination CBZ in real samples with satisfactory results.
